# Supplementary material for: Assessing the Nutritional Value of Root and Tuber Crops from Bolivia and Peru
Source: Foods. 2019 Oct 23;8(11):526. doi: 10.3390/foods8110526 (PMC6915682; doi:10.3390/foods8110526)
Supplement: Supplementary file 1 [file foods-08-00526-s001.pdf]

Supplementary Table:

Assessing the nutritional value of root and tubers crops from Bolivia and Perú.

L.A. Choquechambi, I.R. Callisaya, A. Ramos, H. Bosque, A. Mújica, S.E.

Jacobsen, M. Sørensen, E.O. Leidi

Landraces of mashua, arracacha and yacon collected in Bolivia and Peru during 2015, 2016 and 2017 with the available data of collection site and altitude (in meters above sea level, m.a.s.l.).

	Crop	Landrace	Site of collection	Altitude (m.a.s.l)	Date of harvest
Bolivia	Mashua	*Chiar* (black)	Cantón Italaque	3,468	14 July 2015
		Kellu (yellow)	Id.		
		Keni kellu (purple- yellow)	Id.		Id.
		Jachir (Yellow, red stripes)	Id.		Id.
		Asuthi (yellow, purple stripes)	Id.		Id.
	Arracacha	Yellow	San Juan de la Miel	1,979	14 July 2017
	Yacon	*Kulli* (purple)	Mocomoco	3,333	Not reported.
		Kulli (id.)	Sorata		
		White	Id.		Id.
Peru	Mashua	*Kellu* (yellow)	Conima, Puno	3,860	25 August 2015
		Ch'ejchi (yellow, black-eyed)	Id.		
	Arracacha	Purple yellow	San Juan del Oro, Puno	1,315	28 August 2015
	Yacon	White	Sandia, Puno	2,993	27 August 2015
